# Need for Representation of Pediatric Patients with Obesity in Clinical Trials

**DOI:** 10.3390/children10101640

**Published:** 2023-09-30

**Authors:** Sherbet Samuels, Jayabharathi Vaidyanathan, Elimika Pfuma Fletcher, Anuradha Ramamoorthy, Rajanikanth Madabushi, Gilbert J. Burckart

**Affiliations:** Office of Clinical Pharmacology, Center for Drug Evaluation and Research, US Food and Drug Administration, Silver Spring, MD 20993, USAelimika.fletcher@fda.hhs.gov (E.P.F.); anuradha.ramamoorthy@fda.hhs.gov (A.R.); rajanikanth.madabushi@fda.hhs.gov (R.M.)

**Keywords:** pediatrics, obesity, clinical trials, diversity, drug dosing, drug development

## Abstract

Clinical trials are an integral aspect of drug development. Tremendous progress has been made in ensuring drug products are effective and safe for use in the intended pediatric population, but there remains a paucity of information to guide drug dosages in pediatric patients with obesity. This is concerning because obesity may influence the disposition of drug products. When pediatric patients with obesity are not enrolled in clinical trials, dosing options for use in this subpopulation may be suboptimal. Reliance on physiological-based dosing strategies that are not informed by evaluation of the pharmacokinetics of the drug product could lead to under- or over-dosing with ensuing therapeutic failure or toxicity consequences. Thus, representation of pediatric patients with obesity in clinical trials is crucial to understand the benefit-risk profile of drug products in this subpopulation. It is important to acknowledge that this is a challenging endeavor, but not one that is insurmountable. Collective efforts from multiple stakeholders including drug developers and regulators to enhance diversity in clinical trials can help fill critical gaps in knowledge related to the influence of obesity on drug disposition.

## 1. Introduction

Conducting clinical trials in the pediatric population is complex due in part to maturational and physiological development across age groups, ethical considerations, and feasibility. Despite these challenges, clinical trials are integral to advance pediatric drug development. In the United States (US), legislative endeavors have substantially increased the number of pediatric drug studies conducted across a wide range of US Food and Drug administration (FDA) therapeutic areas [1,2]. Since 1998, over 1000 drug product labeling have been updated with pediatric-specific information about the drug product’s effectiveness, safety, or use [3]. Obesity further compounds the difficulties with pediatric drug development because in addition to growth and maturation, obesity could influence the disposition of drug products [4,5,6].

Obesity is a global public health challenge with far-reaching health consequences. Underrepresentation of patients with obesity in clinical drug development trials can lead to limitations in generalizability of the clinical trial findings and gaps in drug product labeling recommendations. Although there is an increase in availability of pediatric therapeutic drug options, there remains a paucity of information to guide drug dosage in the subpopulation of pediatric patients with obesity. A review of drug product labeling for 89 products found a lack of information about the pediatric dosage for patients with obesity and only four of the drug product labels contained information related to the effect of body mass index (BMI) on the pharmacokinetics of the drug product [7]. This is concerning because the prevalence of obesity in the US among individuals ages 2 to 19 years old was 19.7% between 2017 and March 2020 [8]. Furthermore, over 100 million children worldwide were considered to have obesity in 2015 [9]. Among middle- and low-income countries, the median prevalence of comorbidities such as hypertension (35.6%), metabolic syndrome (26.9%), non-alcoholic fatty liver disease (47.5%), and dyslipidemia (43.5%) in the pediatric population with obesity further amplifies the public health concerns [10]. Herein, we discuss the importance of representation of pediatric patients with obesity in clinical trials, highlight regulatory initiatives in this area, and propose some strategies to support clinical trial diversity.

## 2. Importance of Representation of Pediatric Patients with Obesity in Clinical Trials

Obesity is a chronic medical condition defined by excess body fat and is associated with many comorbidities including increased risks of type 2 diabetes, cardiovascular disease, liver disease, and metabolic syndrome in the pediatric population [11]. Additionally, obesity may affect several systems in the body including hepatic blood flow, plasma proteins, gastric permeability, and enzyme activity [6]. Obesity status is classically determined by BMI derived from kilograms of weight divided by height in meters squared (kg/m^2^) [12,13]. The Center for Disease Control and Prevention (CDC) published references for determining obesity based on BMI measures. Adult individuals with a BMI equal to or greater than 30 kg/m^2^ are considered to have obesity [13]. Classification of obesity in the pediatric population is often based on the CDC growth chart. Pediatric individuals with a BMI equal to or greater than the 95th percentile among those of the same age and sex are considered to have obesity [12].

The CDC acknowledged that the use of BMI as a surrogate measure of obesity is not without limitations [14]. Height and maturation in the pediatric population could influence BMI. Additionally, BMI does not measure body composition and does not differentiate between fat and fat-free mass (muscle mass or bone mass) [14]. Other measures of adiposity include waist-to-height ratio, waist circumference, skinfold thickness, and dual energy x-ray absorption. However, these measures also have limitations in terms of standardization, availability, or expense [14]. Another part of the conundrum with obesity in the pediatric population is assessing the obesity phenotype. The distinction of metabolically healthy obesity from metabolically unhealthy obesity hinges on the cardiometabolic factors that are assessed, such as ethnicity and maturation in reference to puberty [15]. More research is needed in this area to better understand how these types of obesity phenotype classifications could affect drug disposition.

**Figure 1:**
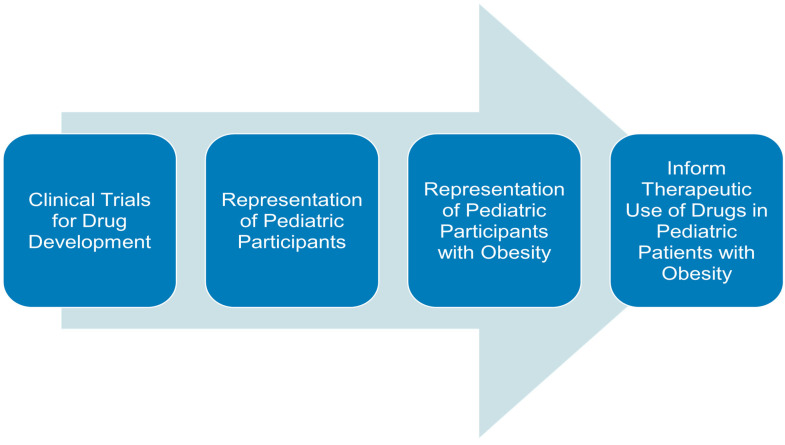
Schematic of the clinical trial representation process that could inform therapeutic use of drugs in pediatric patients with obesity.

In general, dosing guidelines for pediatric patients are often based on the physiological characteristics of the patients (age, body weight, body surface area) or allometric scaling [16,17]. A review of pediatric dosing for drug products commonly used in the prehospital setting found deviations from national guidelines in reference to weight-based dosing [18]. Among the drugs reviewed, dosing of lorazepam (21.2%) and diazepam (19.5%) had the lowest consistency with national guidelines. Most of the deviations from national guidelines related to underdosing. Variations in dosing protocols could have played a role in the deviations from national guidelines [18]. Furthermore, it is uncertain how the pediatric dosing guidelines are developed. In the absence of the pharmacokinetic evaluation of drug products, general pediatric dosing guidelines may not result in optimal therapeutic dosing. Furthermore, a variety of obesity-related physiological changes combined with the physiochemical properties of a drug could affect the pharmacokinetics of the specific drug product (e.g., volume of distribution and clearance) [4,5,11,19,20]. In these cases, dosage adjustments may be necessary to reduce the risk of therapeutic failure or drug toxicities [4]. Thus, it is prudent for patients with obesity to be represented in clinical drug development trials to understand the influence of obesity on the disposition of individual drug products and to generate evidence to optimize dosage in this subpopulation (Figure 1).

A review of randomized controlled trials of various types of cancers in adults, for which obesity is a risk factor, found eligibility and enrollment information about patients with obesity were scant [21]. Although enrollment criteria did not exclude participants based on BMI, most of the trial reports did not identify the proportion of participants who had obesity [21]. Similar to the adult population, there has been an increase in attention to the lack of information in drug product labeling about dosage recommendations related to obesity in the pediatric population [7,11,20].

Under the US regulatory framework, the drug product labeling is used to communicate the essential information about how to use a drug product effectively and safely [22]. Information regarding obesity may be included in the drug product labeling based on the availability of sufficient data about the use of the drug in this specific subpopulation. For example, the drug product labeling for midazolam in sodium chloride injection notes that the mean half-life of midazolam was greater in subjects with obesity compared to subjects without obesity [23]. The drug product labeling recommends the midazolam dose in pediatric patients with obesity be based on ideal body weight (IBW) [23]. Similarly, the drug product labeling for remifentanil hydrochloride, which is approved for use in both adults and pediatric patients, recommends that the starting doses in patients with obesity (greater than 30% over their IBW) be based on IBW [24]. The remifentanil hydrochloride drug product labeling also notes that caution is required with use of potent opioids in patients with morbid obesity due to alterations in cardiovascular and respiratory physiology [24].

Given the prevalence of obesity in the pediatric population, it is pragmatic to expect that once a drug is approved for a pediatric indication, it is likely to be used in the subpopulation with obesity. The fundamental driver for inclusion of information in the drug product labeling is the availability of data. If pediatric patients with obesity are underrepresented in clinical trials, adequate information may not be available to inform optimal use of the drug products and clinical decision-making in this subpopulation. While the above examples reflect the inclusion of obesity-related information in some drug product labeling, most labeling do not contain obesity-related dosage information [7]. Enhanced diversity in clinical trials that ensures the representation of pediatric patients with obesity can help fill this gap.

## 3. Regulatory Initiatives Related to Clinical Trial Diversity and Obesity

Ensuring diversity in clinical drug development trials is a challenge for the pharmaceutical industry, researchers, regulators, and the public health community. Multiple factors may influence clinical trial participation. For example, the underrepresentation of pediatric patients with obesity in clinical trials may be intertwined with the underrepresentation of racial and ethnic minorities in that racial and ethnic minorities have a higher prevalence of obesity. In the US, Hispanic (26.2%) and non-Hispanic Black (24.8%) had a higher prevalence of obesity among individuals ages 2 to 19 years old compared to those who were non-Hispanic White (16.6%) and non-Hispanic Asian (9.0%) [8]. Of note, Hispanic and Black ethnic and racial groups are often underrepresented in clinical trials [25].

In the regulatory space, considerable efforts have been embarked upon to enhance diversity in clinical drug development trials (Table 1).

The FDA initiatives include providing recommendations for approaches to help ensure diversity in clinical drug development trials as well as data collection, analysis, reporting, and communication [35,36]. A 2022 FDA guidance document recommends the development of a plan among other strategies to bolster inclusion of participants from underrepresented racial and ethnic groups in clinical drug development trials [27]. Another FDA guidance document focuses on strategies to enhance participation from an underrepresented population in clinical drug development trials, including those in the higher weight range [28]. The FDA’s guidance document highlighted considerations for factors that may impede participation and encouraged the adaptation of enrollment and retention practices that enhance inclusiveness [28]. One of the key recommendations to enhance diversity in clinical trials is through broadening eligibility criteria without compromising the safety of trial participants with a keen focus on inclusive practices, trial designs, and methodological considerations [28]. In addition to addressing clinical drug development trial diversity, the FDA has also issued guidance documents that include recommendations for obesity to be considered in pediatric drug development [17,30].

In pediatrics as well as in adults, the cornerstone for managing obesity is lifestyle modifications including changes to dietary intake, physical activity, and other behaviors. However, under certain circumstances, drug therapeutic options may be considered, for example, treatment with a weight-management drug product can be considered for pediatric or adult patients with obesity who have weight-related comorbidities where lifestyle modifications have not been successful, and the benefits of treatment outweigh the risks [29]. The FDA has issued a guidance for the development of weight-management drug products, which encourages inclusion of participants from diverse demographic, ethnic, and racial groups with a high prevalence of obesity [29]. This guidance document recommends that the pharmacokinetics of the drug be evaluated in participants with a wide range of BMIs to identify the most appropriate drug dosage [29]. The goal of weight-management drug therapy, emphasized in the FDA’s guidance document, shows improvements in biomarkers, such as hemoglobin A1C, blood pressure, and lipids, to reduce the risks of morbidity and mortality [29]. In reference to the pediatric population, the guidance document recommends a medical assessment to identify genetic or endocrinologic causes of obesity, as well as to screen for comorbidities including dyslipidemia, hypertension, and glucose intolerance [29].

Other FDA initiatives in this space includes engaging stakeholders. In November 2022, a scientific workshop was held to discuss approaches to bridge the safety and effectiveness of drugs to populations with obesity [37]. In this forum, stakeholders from industry, academia, and regulatory agencies discussed considerations for evaluating the effect of obesity on the safety and effectiveness of drug products. The workshop discussions focused on adopting innovative solutions to improve obesity-related information in reference to drug response.

## 4. Considerations for Strategies to Support Clinical Trial Diversity

The FDA initiatives have laid the foundation upon which further multifaceted approaches can be built to help increase understanding of the benefits and risks associated with a drug product when used across diverse populations. Diversity in clinical drug development trials helps with translating results that are more applicable to patients in the real-world setting. In the context of pediatric obesity, continued engagement among stakeholders throughout the lifecycle of a drug, including during development and post-marketing phases, can help identify and address barriers that impede clinical trial participation and contribute to the dearth of obesity-related information in drug product labeling. Considerations for strategies to improve representation of patients with obesity in clinical trials can be combined with broader diversity efforts.

Employing measures to build trust in communities where a lack of trust may be a barrier to clinical trial participation, such as including minority communities with a high prevalence of the pediatric population with obesity, can help broaden interest in clinical trial participation. One of the key strategies to help build trust in marginalized communities is through partnerships with community members to plan and conduct trials [38]. At the patient level, providing an explanation of the reasonably foreseeable risks, as well as the benefits of participating in a clinical trial and recognizing autonomy in making informed decisions about trial participation, can help build trust. Additionally, transparency and clear communication about drug development and clinical trial activities can contribute to building trust in communities and with specific patient populations, such as those with obesity.

Stakeholder engagement plays an important role in enhancing clinical trial diversity. In addition to engagements with regulators, drug developers, and researchers to identify innovative strategies to enhance clinical trials, engagement at the community level can help support diversity in clinical trials [28]. Through engagement with patient advocacy groups such as community leaders and community members, issues with language barriers, health literacy, clinical trial misconceptions, and social stigmas can be identified and addressed to broaden clinical trial participation. Engagement at the community level can also help to build trust and support for clinical trials. At the community level, engagement can go beyond the confines of clinical trials and help support and sustain the overall health and wellbeing of community members.

Health care professionals are at the helm of patient engagement, clinical trial recruitment, and clinical trial enrollment activities. Given their central role, the training and education of health care professionals is essential to address the underrepresentation of patients with obesity in clinical trials. It is important to ensure that health care professionals understand clinical trial enrollment and are aware of strategies to improve diversity in participation. Likewise, understanding the reasons why health care professionals may be hesitant to offer clinical trial participation opportunities to their patients with obesity is critical to change the current situation. For example, assessments of health care professionals’ underlying assumptions about patients with obesity and the presence of comorbidities that may be a barrier to recruitment for clinical trials may be addressed through training, education, and trial designs. Ultimately, enhancement of clinical trial diversity does not preclude risk-benefit assessments to ensure that safety standards for clinical trial participants are maintained [28].

Critical evaluation of clinical trial eligibility criteria is a crucial aspect of reducing barriers to clinical trial participation. Restrictive eligibility criteria may unnecessarily exclude patients with obesity from participation in clinical trials and create gaps in knowledge about the safety and effectiveness of drug products for this subpopulation. When enrollment criteria may be an issue, measures can be employed to ensure trial designs and methodologies meet the goals of the drug development phase. For example, a critical assessment of the issues that can be addressed to broaden clinical trial enrollment include (a) whether clinical trial exclusion criteria related to specific BMI measures serves a role of mitigating known risks that outweighs benefits, (b) whether criteria are merely a carry-over from previous phases of drug development, (c) whether criteria are based on templates utilized across trials, or (d) whether clinical trial criteria can be reduced based on available data about the specific drug [28].

Furthermore, a more patient-centric approach to designing and conducting clinical trials with consideration given to the patient population and the practicalities of participation can help facilitate participation. Attention to clinical trial site locations, frequency of trial site visits, options for reasonable accommodations, availability of mobile health care professions to monitor key protocol elements, and measures to reduce financial or other burdens can help to address logistical issues that could limit the clinical trial participation [28]. Additionally, the leveraging of existing data and other innovative approaches like modeling and simulation can help support development of drug products and inform clinical trial designs.

While the impact of these FDA initiatives is unknown as yet, the expectation is that these efforts, combined with multifaced strategies, will help advance the field of drug development across diverse populations. Nevertheless, more concerted efforts from multiple stakeholders, including drug developers, regulators, researchers, clinicians, and patient advocacy groups, can help fill critical gaps in knowledge regarding the influence of obesity on individual drug product disposition. Enhanced diversity in clinical drug development trials that ensure the representation of patients with obesity can inform therapeutic options across the pediatric population and will help to ensure that drug development can provide accurate dosing guidelines for pediatric patients with obesity.

## 5. Conclusions

Despite the tremendous progress in advancing pediatric drug development, pediatric patients with obesity are still underrepresented in clinical trials, leading to gaps in knowledge about the benefit-risk profile of specific drug products in this subpopulation. The regulatory framework in the US allows for communication of obesity-related information in the drug product labeling, but sufficient data is vital to inform labeling recommendations. Regulator initiatives include the issuance of guidance documents and stakeholder engagement through workshops that emphasize strategies to enhance clinical trial diversity. While endeavors such as these can help advance drug development, more work involving multifaced approaches can further improve the representation of patients with obesity in clinical trials and fill gaps in knowledge to better inform optimal drug product use in the pediatric subpopulation with obesity.

## Figures and Tables

**Table 1:** Examples of FDA guidance documents related to the inclusion of patients with obesity and/or underrepresented racial and ethnic populations in clinical drug development trials.

Guidance for Industry * [26]	Overview and Specific Obesity, Race, or Ethnicity-Related Information
*General Drug Development*	
General Clinical Pharmacology Considerations for Pediatric Studies of Drugs, Including Biological Products (Draft, 2022) [17]	Considerations for characterizing dosing and safety of drugs for pediatric use: Assess the effect of obesity on drug disposition and response Obtain covariates and phenotype data (race and ethnicity, calculated BMI, body weight, height)
Diversity Plans to Improve Enrollment of Participants From Underrepresented Racial and Ethnic Populations in Clinical Trials (Draft, 2022) [27]	Recommendations for inclusion of participants from underrepresented racial and ethinic groups in clinical trials
Enhancing the Diversity of Clinical Trial Populations—Eligibility Criteria, Enrollment Practices, and Trial Designs (Final, 2020) [28]	Approaches to increase enrollment of underrepresented populations in clinical trials: Consider characteristics of the study population (extremes of the weight range, race and ethnicity, location) Ensure baseline characteristics of participants are reflective of the target patient population
Developing Products for Weight Management (Draft, 2007) [29]	Recommendations for developing development of drugs and therapeutic biologics for weight management
*Specific Therapeutic Areas*	
Development of Anti-Infective Drug Products for the Pediatric Population (Final, 2021) [30]	General recommendations for developing anti-infective drug products: Consider body weight and body surface area when determining the cohorts to be studied Assess for the effect of obesity on dose selection
Establishing Effectiveness and Safety for Hormonal Drug Products Intended to Prevent Pregnancy (Draft, 2019) [31]	Recommendations for designing clinical trials for hormonal drug products to prevent pregnancy: Enrollment criteria should include women with obesity Subgroup analysis of efficacy in women with obesity should be prespecified in the analysis plan Insufficient data in women with obesity may result in limitation of use
Noncirrhotic Nonalcoholic Steatohepatitis With Liver Fibrosis: Developing Drugs for Treatment (Draft, 2018) [32]	Recommendations for the development of drugs for noncirrhotic nonalcoholic steatohepatitis (NASH) with liver fibrosis: The proportion of patients in clinical trials with comorbidities such as obesity or type 2 diabetes should be reflective of the intended patient population
Anthrax: Developing Drugs for Prophylaxis of Inhalational Anthrax (Final, 2018) [33]	Recommendations for development of drugs for prophylaxis of inhalational anthrax: Considerations for clinical pharmacology evaluation include obtaining pharmacokinetic data from patients with obesity or morbid obesity
*Drug Product Labeling*	
Clinical Pharmacology Section of Labeling for Human Prescription Drug and Biological Products—Content and Format (Final, 2016) [34]	Recommendations for presenting clinical pharmacology information in prescription drug labeling (i.e., CLINICAL PHARMACOLOGY section): Include race or ethnicity-related pharmacokinetic analysis results Include obesity-related pharmacokinetic analysis results

* FDA-issued guidance documents are available to the public at https://www.fda.gov/regulatory-information/search-fda-guidance-documents (accessed on 27 August 2023).

## Data Availability

Not applicable.
